# Numerical assessment of heat and mass transportation in $\upgamma {\mathrm{Al}}_{2}{\mathrm{O}}_{3}{-}{\mathrm{H}}_{2}\mathrm{O}/{\mathrm{C}}_{2}{\mathrm{H}}_{6}{\mathrm{O}}_{2}$ nanofluids influenced by Soret and Dufour effects

**DOI:** 10.1038/s41598-022-07453-4

**Published:** 2022-03-07

**Authors:** Tamour Zubair, Muhammad Usman, Kottakkaran Sooppy Nisar, Ilyas Khan, H. Y. Zahran, Abdulrazak H. Almaliki

**Affiliations:** 1 School of Mathematical Sciences, Peking University, Beijing, 100871 China; 2 Department of Mathematics, National University of Modern Languages (NUML), Islamabad, 44000 Pakistan; 3 Department of Mathematics, College of Arts and Sciences, Prince Sattam Bin Abdulaziz University, Wadi Aldawaser, 11991 Saudi Arabia; 4 Department of Mathematics, College of Science Al-Zulfi, Majmaah University, Al-Majmaah, 11952 Saudi Arabia; 5 Laboratory of Nano-Smart Materials for Science and Technology (LNSMST), Department of Physics, Faculty of Science, King Khalid University, P.O. Box 9004, Abha, Saudi Arabia; 6 Research Center for Advanced Materials Science (RCAMS), King Khalid University, P.O. Box 9004, Abha, 61413 Saudi Arabia; 7 Nanoscience Laboratory for Environmental and Biomedical Applications (NLEBA), Metallurgical Lab.1, Department of Physics, Faculty of Education, Ain Shams University, Roxy Cairo, 11757 Egypt; 8 Department of Civil Engineering, College of Engineering, Taif University, P.O. Box 11099, Taif, 21944 Saudi Arabia

**Keywords:** Mathematics and computing, Applied mathematics, Computational science, Software

## Abstract

The current flow model is dedicated to capture the role of Dufour and Soret on heat and mass transmission of rotating flow of $\upgamma {\mathrm{Al}}_{2}{\mathrm{O}}_{3}-{\mathrm{C}}_{2}{\mathrm{H}}_{6}{\mathrm{O}}_{2}$ and $\upgamma {\mathrm{Al}}_{2}{\mathrm{O}}_{3}-$water nanoparticles due to exponential stretching under the action of thermal radiation, magnetic, and Eckert numbers. The problem is modelled in terms if partial differential equations (PDEs) with associated physical conditions. The ordinary differential equations (ODEs) are obtained via suitable transformations. The reduced nonlinear ODEs set is tackled via a new scheme. We suggested significant improvements in the traditional technique and further formulated an extended version of wavelets scheme-based Chebyshev polynomials thoughts. The detailed procedure of the wavelet scheme and flow chart are provided. To validate the numerical outcomes; a comparative study with numerical technique RK (order-4) is also provided. Furthermore; numerical consequences of velocity, concentration and temperature profiles are further examined using several plots. The graphical plots, compared and convergence analyses are endorsing that our proposed modifications are worthy. Velocities profiles in view of $\gamma {\mathrm{Al}}_{2}{\mathrm{O}}_{3}-{\mathrm{C}}_{2}{\mathrm{H}}_{6}{\mathrm{O}}_{2}$ nanofluid are lower than the $\gamma {\mathrm{Al}}_{2}{\mathrm{O}}_{3}{-}{\mathrm{H}}_{2}\mathrm{O}$ nanofluid. Temperature and concentration profiles are dominant when $\gamma {\mathrm{Al}}_{2}{\mathrm{O}}_{3}{-}{\mathrm{H}}_{2}\mathrm{O}$ nanofluid is considered.

## Introduction

The rotating flows includes a lot of applications in engineering and geophysics food making, chemical process, magma flow, rotor–stator technology, rotational of machinery, centrifugal filtration, flow circulations (anti-cyclonic), tornadoes and hurricanes. The mentioned situations are endorsing scientists to study the rotational flow phenomena in view of stretching surface. Wang^1^ did the pioneer contribution in this regard. He provided a perturbation solution for his proposed model and analyzed the velocity. Crane^2^ captured the study of motion in fluid particles induced by moveable surface. Takhar and Nath^3^ discussed rotating flow due to rotational movement of sheet. Andersson^4^ studied the slip flow past a stretching surface. He assumed the Newtonian slip-flow fluid towards stretching surface and used an analytical approach to obtain an exact solution for suggested model. In a revolving fluid, an investigation of rotational flow occurred due to stretching surface is studied by^5^. El Aziz^6^ inspected the impact of thermal energy considering Blowing/suction under the action of magnetic field. Kumari et al.^7^ discussed the simulations of heat in power-law liquid. Govardhan et al.^8^ scrutinized the impression of rotating flow occurred due to porous surface.

The composite study of mass and heat is vital factor in several fields like power generation, air conditioning, space heating and air conditioning called engineering applications. The transportation of species is made vital effects in fields of engineering and industries. The useful applications of mass are food making, drug transport, oil delivery and certain medicines (blood) respectively. Such vital applications are motivated the scientists and engineers to capture phenomenal action of mass and heat in view of experimentally and theoretically. Some latest work related to mass and heat is found. The related work of this field is mention in^9–14^.

Recent developments in nano-technology have determined a latest area for the enhancement in the performance of heat called nanoparticles. The suspension of nano-structures in base fluids (water, oil, kerosene) provides a better heat transfer and enhanced the convection and conduction coefficients. These machinimas are very useful to improve conductivity of heat energy. The applications of nano-particles for performance of heat are solar heating, refrigerators, cooling process, heat exchange, drag reduction, storage (thermal energy) and drag reduction respectively.

The suspension of various kinds of nanoparticles in base fluid is used by various authors. The iron based nanoparticles inside the fluids (Ferro-fluids) are quite suitable because their thermo-physical characteristics can be controlled by means of magnetic field. An excellent literature related to Ferro-fluids can be find in^15–18^. Usman et al.^19^ studied enhancement in motion of fluid particles using nanoparticles towards channel. Mohyud-Din et al.^20^ examined the motion in nanoparticles considering Marangoni convection and thermal radiation. Mushtaq et al.^21^ discussed the rotational motion in nanofluids caused by sheet.

The domain of nanofluids is gained a vital importance due to its various applications. A noteworthy study related to this domain is available in literature. Many models developed after the Choi^22^ contribution. The Xue^23^, Buongiorno^24^ and Tiwari Das^16^ models are some major contributions. Sheremet et al.^25,26^ discussed the Tiwari Das approach in porous square cavity inserted by nanoparticles and the role of thermal dispersion in porous wavy-walled (cavity) with natural convection is discussed. This domain has been studied by many scholars by considering various aspects^27–32^.

Sheikholeslami et al.^27,28^ studied the enrichments in heat (energy) with nanoparticles under the action of magnetic number and radiation (thermal). Nazir et al.^29^ analyzed behavior of Casson liquid inserting nanoparticles called $Cu$ and $Ag$ involving the effect of ion-slip and Hall currents over a melting surface. They discussed viscous dissipation (VD) and variable magnetic field (MF) in flow model via FEM approach. Rashidi et al.^30^ examined the process of entropy production in a particular shape of tube heat transmission via nanofluids. Usman et al.^31^ analyzed heat in view of unsteady model via analytical approach. Khan et al.^32^ captured flow situation of second grade liquid including Dufour and Soret influences past melting channel. A detailed review regarding the applications of heating or cooling processes using different types of nanomaterials is given in^33^.

Motivation of the present analysis is to investigate mass and heat energy including Dufour and Soret influences in rotational flow under nanofluids ($\upgamma {\mathrm{Al}}_{2}{\mathrm{O}}_{3}-\mathrm{EG} and\upgamma {\mathrm{Al}}_{2}{\mathrm{O}}_{3}-\mathrm{water}$) towards melting surface. The current model is an extension of^21^ wherein we observed the study of magnetic, Eckert numbers and thermal radiation via melting surface. The modeling and geometry of mathematical model presented. The set of ODEs obtained via suitable transformations whereas ODEs are simulated via new approach called modified Chebyshev wavelets method. Previously, this mentioned technique is operated by several authors but current solution approach is used first time in fluid model. The work related to traditional Chebyshev wavelets method can be find in^34–36^. A worthy comparison between outcomes obtained also presented^37–46^. The graphical results are captured with variation in parameters. The graphical plots, comparison and convergence analysis are endorsing that our modification is very worthy.

## Formulation of flow model

**Figure 1:**
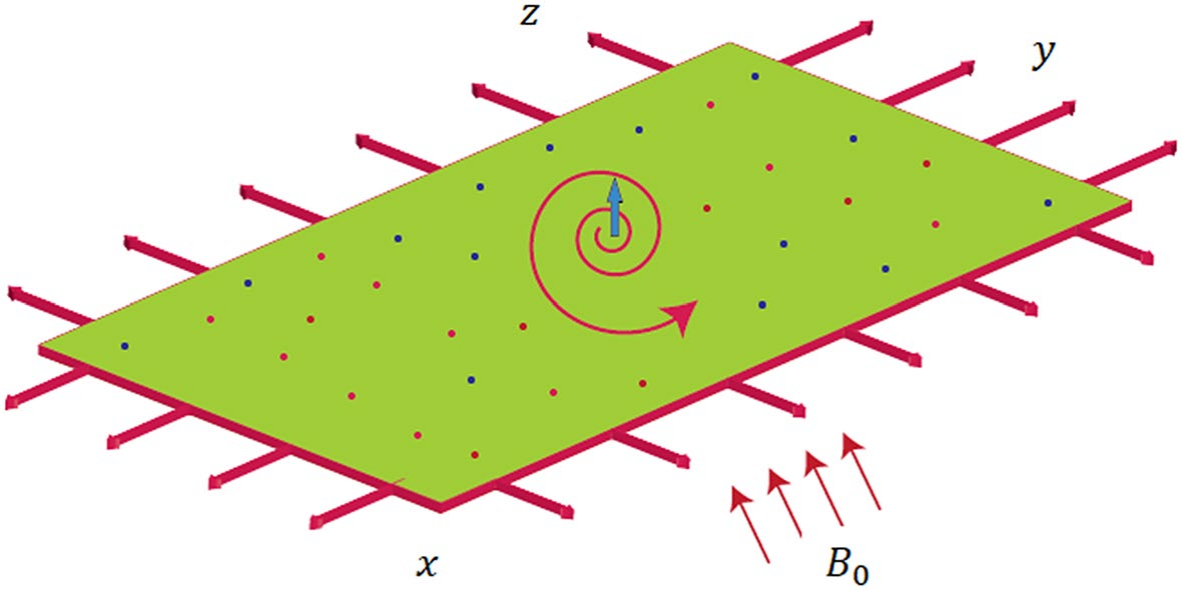
The flow behavior of nanoparticles.

The physical aspects of mass and heat transmission for rotating nanofluid with an velocity (angular) $\uplambda =\Omega {\mathrm{e}}^{-x/L}$ around $z$-axis where $\Omega$ and $L$ are average form of velocity and characteristic distance respectively, we ponder a viscous, incompressible, radiative (significant radiations effects) and steady fluid over a common sheet (elastic nature) which is widening exponentially along $x-$ axis and the fluid is surviving with $z=0.$ ${T}_{w}$ and ${C}_{w}$ are considered as temperature (constant) and concentration (constant) at the surface respectively and in the similar context, ${T}_{\infty }$ and ${C}_{\infty }$ are the temperature (constant) and concentration (constant) of fluid at ambient position respectively. Magnetic field of constant nature is applied and chemical reaction of first order is implemented to the system under the action of Dufour and Soret influences. Due to this context, the magnetic field is induced which is consider negligible owing to small value of Reynolds number. It is very important to nominate that two categories of nano particles i.e. $\upgamma {\mathrm{Al}}_{2}{\mathrm{O}}_{3}-$ethylene glycol and $\upgamma {\mathrm{Al}}_{2}{\mathrm{O}}_{3}-$water are used for the generated system. Under all the considered assumption, the mathematical form of governing rules is simulated as^5,21,41^:

1 $$\frac{\partial u}{\partial x}+\frac{\partial v}{\partial y}+\frac{\partial w}{\partial z}=0,$$

2 $$u\frac{\partial u}{\partial x}+v\frac{\partial u}{\partial y}+w\frac{\partial u}{\partial z}-2\Omega v={\nu }_{nf}\frac{{\partial }^{2}u}{\partial {z}^{2}}-\frac{{\sigma }_{nf}}{{\rho }_{nf}}{B}_{0}^{2}u,$$

3 $$u\frac{\partial v}{\partial x}+v\frac{\partial v}{\partial y}+w\frac{\partial v}{\partial z}+2\Omega u={\nu }_{nf}\frac{{\partial }^{2}v}{\partial {z}^{2}}-\frac{{\sigma }_{nf}}{{\rho }_{nf}}{B}_{0}^{2}v,$$

4 $$\frac{{\left(\rho {c}_{p}\right)}_{nf}}{{\left(\rho {c}_{p}\right)}_{f}}\left(u\frac{\partial T}{\partial x}+v\frac{\partial T}{\partial y}+w\frac{\partial T}{\partial z}\right)=\frac{{k}_{nf}}{{\left(\rho {c}_{p}\right)}_{f}}\frac{{\partial }^{2}T}{\partial {z}^{2}}+\frac{D{k}_{T}}{{c}_{s}{\left({c}_{p}\right)}_{f}}\frac{{\partial }^{2}C}{\partial {z}^{2}}+\frac{{\mu }_{nf}}{{\left(\rho {c}_{p}\right)}_{f}}\left({\left(\frac{\partial u}{\partial z}\right)}^{2}+{\left(\frac{\partial v}{\partial z}\right)}^{2}\right)+\frac{16{\sigma }^{*}{T}_{\infty }^{3}}{3{k}^{*}{\left(\rho {c}_{p}\right)}_{f}}\frac{{\partial }^{2}T}{\partial {z}^{2}},$$

5 $$u\frac{\partial C}{\partial x}+v\frac{\partial C}{\partial y}+w\frac{\partial C}{\partial z}=D\frac{{\partial }^{2}C}{\partial {z}^{2}}+\frac{D{k}_{T}}{{T}_{m}}\frac{{\partial }^{2}T}{\partial {z}^{2}}-{k}_{1}\left(C-{C}_{\infty }\right),$$

where $u, v, w, T, C, {\nu }_{nf},$ ${\sigma }_{nf}, {\rho }_{nf}, {B}_{0}, {\left({c}_{p}\right)}_{nf}, {\left({c}_{p}\right)}_{f}, D{, k}_{T}, {k}_{nf}, {k}^{*}, {T}_{m}, {\sigma }^{*}, {k}_{1}, {C}_{s}$ are entitled as $x, y, z$-components of velocity, temperature, concentration of solute, kinematics viscosity for nano-fluid, electrical conductivity of nano-fluid, density of nano-fluid, magnetic field component, heat capacity for nano-fluid, heat capacity for fluid, mass diffusion coefficient, thermal diffusion ratio, thermal conductivity for nano-fluid, mean absorption, mean temperature, Steffen Boltzmann constant, rate of chemical reaction and the susceptibility of concentration respectively. The flow configurations is sketched by Fig. 1.

The boundary conditions (BC’s) are connected with defined problem in Eqs. (1–5) are given as:

6 $$u={U}_{w}\left(x\right)=U{\mathrm{e}}^{x/L },v=w=0,T={T}_{w}, C={C}_{w}, \quad for \quad z=0,$$

7 $$u,v\to 0,T\to {T}_{\infty }, C\to {C}_{\infty }, \quad for \quad z\to \infty$$

We arrived by means of two different models (for $\gamma {\mathrm{Al}}_{2}{\mathrm{O}}_{3}{-}{\mathrm{H}}_{2}\mathrm{O}$ and $\gamma {\mathrm{Al}}_{2}{\mathrm{O}}_{3}-{\mathrm{C}}_{2}{\mathrm{H}}_{6}{\mathrm{O}}_{2}$) of nanofluids along with the following relations^37^:

8 $$\frac{{\rho }_{nf}}{{\rho }_{f}}=\left(1-\phi +\frac{{\rho }_{s}}{{\rho }_{f}}\phi \right),\frac{{\sigma }_{nf}}{{\sigma }_{f}}=\left(1+\frac{3\left(\sigma -1\right)\phi }{\left(\sigma +2\right)-\left(\sigma -1\right)\phi }\right),\frac{{\left(\rho {c}_{p}\right)}_{nf}}{{\left(\rho {c}_{p}\right)}_{f}}=\left(1-\phi +\frac{{\left(\rho {c}_{p}\right)}_{s}}{{\left(\rho {c}_{p}\right)}_{f}}\phi \right),$$

where $\phi , {\rho }_{f}, {\rho }_{s}$ are solid volume fraction, fluidic density and solid density respectively. Furthermore, for nano-fluid required expressions like $\mu$, $k$ and $Pr$ are as follow^38,39^:

9 $$\left\{\begin{array}{l}{\mu }_{nf}={\mu }_{f}\left(123{\phi }^{2}+7.3\phi +1\right), \quad for \quad \gamma A{\mathrm{l}}_{2}{\mathrm{O}}_{3}{-}{\mathrm{H}}_{2}O \\ {\mu }_{nf}={\mu }_{f}\left(306{\phi }^{2}-0.19\phi +1\right), \quad for \quad \gamma A{\mathrm{l}}_{2}{\mathrm{O}}_{3}-{\mathrm{C}}_{2}{\mathrm{H}}_{6}{\mathrm{O}}_{2}\end{array}\right.$$

10 $$\left\{\begin{array}{l}{k}_{nf}={k}_{f}\left(4.97{\phi }^{2}+2.72\phi +1\right), \quad for \quad \gamma A{\mathrm{l}}_{2}{\mathrm{O}}_{3}{-}{\mathrm{H}}_{2}O \\ {k}_{nf}={k}_{f}\left(28.905{\phi }^{2}+2.8273\phi +1\right), \quad for \quad \gamma A{\mathrm{l}}_{2}{\mathrm{O}}_{3}-{\mathrm{C}}_{2}{\mathrm{H}}_{6}{\mathrm{O}}_{2}\end{array}\right.$$

11 $$\left\{\begin{array}{l}{\mathrm{Pr}}_{nf}={\mathrm{Pr}}_{f}\left(82.1{\phi }^{2}+3.9\phi +1\right), \, for \, \gamma A{\mathrm{l}}_{2}{\mathrm{O}}_{3}{-}{\mathrm{H}}_{2}O \\ {\mathrm{Pr}}_{nf}={\mathrm{Pr}}_{f}\left(254.3{\phi }^{2}-3.0\phi +1\right). \quad for \quad \gamma A{\mathrm{l}}_{2}{\mathrm{O}}_{3}-{\mathrm{C}}_{2}{\mathrm{H}}_{6}{\mathrm{O}}_{2}\end{array}\right.$$

The desired transformations of current work are:

12 $$\left\{\begin{array}{l}u=U{\mathrm{e}}^\frac{x}{L}{F}^{^{\prime}}\left(\aleph \right), v=U{\mathrm{e}}^\frac{x}{L}G\left(\aleph \right), w=-\sqrt{\frac{{\nu }_{f}U}{2L}}{\mathrm{e}}^\frac{x}{2L} \left[F\left(\aleph \right)+\aleph {F}^{^{\prime}}\left(\aleph \right)\right],\\ \theta \left(\aleph \right)=\frac{T-{T}_{\infty }}{{T}_{w}-{T}_{\infty }},\beta \left(\aleph \right)=\frac{C-{C}_{\infty }}{{C}_{w}-{C}_{\infty }}, \aleph =z\sqrt{\frac{U}{2{\nu }_{f}L}}{\mathrm{e}}^\frac{x}{2L}.\end{array}\right.$$

Using Eqs. (8)–(12) into defined problem Eqs. (1)–(7), once obtained the following:

For γAl_2_O_3_–H_2_O:

13 $$\begin{aligned}&\left(1+7.3\phi +123{\phi }^{2}\right){F}^{{{\prime}}{{\prime}}{{\prime}}}\left(\aleph \right)+\left(1-\phi +\frac{{\rho }_{s}}{{\rho }_{f}}\phi \right)\left(F\left(\aleph \right){F}^{{{\prime}}{{\prime}}}\left(\aleph \right)-2{{F}^{{\prime}}}^{2}\left(\aleph \right)+4\lambda G\left(\aleph \right)\right)\\&\quad-\left(1+\frac{3\left(\sigma -1\right)\phi }{\left(\sigma +2\right)-\left(\sigma -1\right)\phi }\right)H{a}^{2}{F}^{{\prime}}\left(\aleph \right)=0,\end{aligned}$$

14 $$\begin{aligned}&\left(1+7.3\phi +123{\phi }^{2}\right){G}^{{{\prime}}{{\prime}}}\left(\aleph \right)+\left(1-\phi +\frac{{\rho }_{s}}{{\rho }_{f}}\phi \right)\left(F\left(\aleph \right){G}^{^{\prime}}\left(\aleph \right)-2{F}^{^{\prime}}\left(\aleph \right)G\left(\aleph \right)-4\lambda {F}^{{\prime}}\left(\aleph \right)\right)\\ &\quad-\left(1+\frac{3\left(\sigma -1\right)\phi }{\left(\sigma +2\right)-\left(\sigma -1\right)\phi }\right)H{a}^{2}G\left(\aleph \right)=0,\end{aligned}$$

15 $$\begin{aligned}&\frac{\left(\left(1+2.72\phi +4.97{\phi }^{2}\right)+R\right)}{{\mathrm{Pr}}_{f}\left(1+3.9\phi +82.1{\phi }^{2}\right)}{\theta }^{{{\prime}}{{\prime}}}\left(\aleph \right)+\left(1-\phi +\frac{{\left(\rho {c}_{p}\right)}_{s}}{{\left(\rho {c}_{p}\right)}_{f}}\phi \right)F\left(\aleph \right){\theta }^{{\prime}}\left(\aleph \right)\\ &\quad+{D}_{f}{\beta }^{{{\prime}}{{\prime}}}\left(\aleph \right)+\left(1+7.3\phi +123{\phi }^{2}\right){Ec}_{x}\left({{F}^{{{\prime}}{{\prime}}}}^{2}\left(\aleph \right)+{{G}^{{\prime}}}^{2}\left(\aleph \right)\right)=0,\end{aligned}$$

16 $${\beta }^{{{\prime}}{{\prime}}}\left(\aleph \right)+ScF\left(\aleph \right){\beta }^{{\prime}}\left(\aleph \right)+SrSc{\theta }^{{{\prime}}{{\prime}}}\left(\aleph \right)-Sc\delta \beta \left(\aleph \right)\left(\aleph \right)=0.$$

For γAl_2_O_3_–C_2_H_6_O_2_:

17 $$\begin{aligned}&\left(1-0.19\phi +306{\phi }^{2}\right){F}^{{^{\prime}}{^{\prime}}{^{\prime}}}\left(\aleph \right)+\left(1-\phi +\frac{{\rho }_{s}}{{\rho }_{f}}\phi \right)\left(F\left(\aleph \right){F}^{{^{\prime}}{^{\prime}}}\left(\aleph \right)-2{{F}^{{\prime}}}^{2}\left(\aleph \right)+4\lambda G\left(\aleph \right)\right)\\ &\quad-\left(1+\frac{3\left(\sigma -1\right)\phi }{\left(\sigma +2\right)-\left(\sigma -1\right)\phi }\right)H{a}^{2}{F}^{{\prime}}\left(\aleph \right)=0,\end{aligned}$$

18 $$\begin{aligned}&\left(1-0.19\phi +306{\phi }^{2}\right){G}^{{^{\prime}}{^{\prime}}}\left(\aleph \right)+\left(1-\phi +\frac{{\rho }_{s}}{{\rho }_{f}}\phi \right)\left(F\left(\aleph \right){G}^{{\prime}}\left(\aleph \right)-2{F}^{{\prime}}\left(\aleph \right)G\left(\aleph \right)-4\lambda {F}^{{\prime}}\left(\aleph \right)\right)\\ &\quad-\left(1+\frac{3\left(\sigma -1\right)\phi }{\left(\sigma +2\right)-\left(\sigma -1\right)\phi }\right)H{a}^{2}G\left(\aleph \right)=0,\end{aligned}$$

19 $$\begin{aligned}&\frac{\left(\left(1+2.8273\phi +28.905{\phi }^{2}\right)+R\right)}{{\mathrm{Pr}}_{f}\left(1-3.90\phi +254.3{\phi }^{2}\right)}{\theta }^{{^{\prime}}{^{\prime}}}\left(\aleph \right)+\left(1-\phi +\frac{{\left(\rho {c}_{p}\right)}_{s}}{{\left(\rho {c}_{p}\right)}_{f}}\phi \right)F\left(\aleph \right){\theta }^{{\prime}}\left(\aleph \right)+{D}_{f}{\beta }^{{^{\prime}}{^{\prime}}}\left(\aleph \right)\\&\quad+\left(1-0.19\phi +306{\phi }^{2}\right){Ec}_{x}\left({{F}^{{^{\prime}}{^{\prime}}}}^{2}\left(\aleph \right)+{{G}^{{\prime}}}^{2}\left(\aleph \right)\right)=0,\end{aligned}$$

20 $${\beta }^{{^{\prime}}{^{\prime}}}\left(\aleph \right)+ScF\left(\aleph \right){\beta }^{{\prime}}\left(\aleph \right)+SrSc{\theta }^{{^{\prime}}{^{\prime}}}\left(\aleph \right)-Sc\delta \beta \left(\aleph \right)=0.$$

All the parameters involved in the model is explained in Table 1. Furthermore, the dimensionless from of boundary conditions related to problem are given as under:

**Table 1:** Tabulation for important symbols and parameters.

Name	Symbol	Unit	Name	Symbols or expression
$u,v,w$	Velocity components	$\mathrm{m}/\mathrm{s}$	Nano fluid	nf
Density	$\rho$	$\mathrm{kg}/{\mathrm{m}}^{3}$	Fluid	f
Temperature	$T$	$\mathrm{K}/\mathrm{C}$	Solid particle	s
Concentration	$C$	$\mathrm{mol}/{\mathrm{m}}^{3}$	Rate of chemical reaction	${k}_{1}$
Electrical conductivity	$\sigma$	$\mathrm{S}/\mathrm{m}$	Volume fraction	$\phi$
Heat capacity	${C}_{p}$	$\mathrm{J}/\mathrm{K}$	$\mathrm{Ethylene glycol}$	$\upgamma {\mathrm{Al}}_{2}{\mathrm{O}}_{3}$
Thermal conductivity	$k$	$\mathrm{W}/\mathrm{mK}$	Hartmann	$H{a}^{2}=\frac{2L{B}_{0}^{2}}{U}{\mathrm{e}}^{-\frac{x}{L}}$
Current density	${\varvec{J}}$	$\mathrm{A}/{\mathrm{m}}^{2}$	Prandtl	$\frac{1}{\mathrm{Pr}}=\frac{{k}_{f}}{{\nu }_{f}{\left(\rho {c}_{p}\right)}_{f}}$
Viscosity	$\mu$	$\mathrm{PI}$	Diffusion	${D}_{f}=\frac{D{k}_{T}}{{c}_{s}{\left({c}_{p}\right)}_{f}{\nu }_{f}}\frac{\left({C}_{w}-{C}_{\infty }\right)}{\left({T}_{w}-{T}_{\infty }\right)}$
Magnetic field	${\varvec{B}}$	Tesla (T)	Eckert	$E{c}_{x}=\frac{1}{{\left({c}_{p}\right)}_{f}}\frac{{U}^{2}}{{T}_{w}-{T}_{\infty }}{\mathrm{e}}^\frac{2x}{L}$
Electric field	$E$	$\mathrm{N}/\mathrm{C}$	Radiation	$R=\frac{16{\sigma }^{*}{T}_{\infty }^{3}}{3{k}^{*}{k}_{f}}$
Gravitational Acceleration	$g$	$\mathrm{m}/{\mathrm{s}}^{2}$	Schmidt	$Sc=\frac{{\nu }_{f}}{D}$
Kinematics viscosity	$\nu$	${\mathrm{m}}^{2}/\mathrm{s}$	Soret	$Sr=\frac{D{k}_{T}}{{\nu }_{f}{T}_{m}}\frac{\left({T}_{w}-{T}_{\infty }\right)}{\left({C}_{w}-{C}_{\infty }\right)}$
Transformed variable	$\aleph$		Chemical reaction numbers	$\delta =\frac{2{k}_{1}L}{U}{\mathrm{e}}^{-\frac{x}{L}}$

$${\rm for} \quad \aleph =0, \quad F\left(\aleph \right)=G\left(\aleph \right)=0,{F}^{{\prime}}\left(\aleph \right)=\theta \left(\aleph \right)=\beta \left(\aleph \right)=1,$$

$${\rm as} \quad \aleph \to \infty , \quad {F}^{{\prime}}\left(\aleph \right)=G\left(\aleph \right)=\theta \left(\aleph \right)=\beta \left(\aleph \right)=0.$$

## Wavelets and Chebyshev wavelets

Chebyshev wavelets of third kind having four influences $k, i, j, M$ defined in the interval [0, 1] is delivered as^36,37^:

$${\mathfrak{B}}_{i,j}\left(\aleph \right)=\left\{\begin{array}{l}{\left(2\right)}^\frac{k}{2} {\tilde{V }}_{j}\left({2}^{k}\aleph +\left(2i+1\right)\right), \frac{i-1}{ {\left(2\right)}^{k-1}}\le \aleph \le \frac{i}{{\left(2\right)}^{k-1}},\\ 0 \, \, else ,\end{array}\right.$$

In above expression ${\tilde{V }}_{j}\left(\aleph \right)=\frac{1}{\sqrt{\pi }}{V}_{j}\left(\aleph \right), j=\mathrm{0,1},\dots ,M-1, i=\mathrm{0,1},\dots ,{2}^{k-1};$ ${V}_{j}\left(\aleph \right)$ is signifies the *j*-th order of third kind (Chebyshev polynomials) is:

$${V}_{1}\left(\aleph \right)=-1+2\aleph ,{V}_{j}\left(\aleph \right)=2\aleph {V}_{j-1}\left(\aleph \right)-{V}_{j-2}\left(\aleph \right), j\ge 2, {V}_{0}\left(\aleph \right)=1.$$

and further;

$${\Psi }_{{i}_{1}}\left(\aleph \right)={\left.{\Psi }_{{i}_{1}}\left(\aleph \right)\right|}_{{\aleph =2}^{k}\aleph -\left(2{i}_{1}-1\right)}$$

Furthermore; we assumed a special form of function $F\left(\aleph \right)$ as:

$$F\left(\aleph \right)=\sum_{i=1}^{\infty }\sum_{j=0}^{\infty }{\xi }_{i,j}{\mathfrak{B}}_{i,j}\left(\aleph \right),$$

where ${\xi }_{ij}={\int }_{0}^{1}F\left(\aleph \right){\mathfrak{B}}_{ij}\left(\aleph \right){\Psi }_{i}\mathrm{d}\aleph ={\langle F\left(\aleph \right),{\mathfrak{B}}_{i,j}\left(\aleph \right)\rangle }_{{L}_{\Psi }^{2}\left[\mathrm{0,1}\right)}.$ Further, with the truncation, we obtained:

$$F\left(\aleph \right)=\sum_{i=1}^{{2}^{k-1}}\sum_{j=0}^{M-1}{\xi }_{ij}{\mathfrak{B}}_{ij}\left(\aleph \right)={\mathcal{G}}^{T}\mathfrak{B}\left(\aleph \right),$$

The detail discussion regarding matrices $\mathcal{G}$ and $\mathfrak{B}\left(\aleph \right)$ can be perceived in^34–37^.

## Solution technique

In this section, the procedure of solution approach called Chebyshev wavelets scheme is used to find the solution of Eqs. (13–20). The solution steps are listed as:

Step 1. The Eqs. (13–16) are simulated as:

21 $$\begin{aligned}&\left(1+7.3\phi +123{\phi }^{2}\right){F}^{{^{\prime}}{^{\prime}}{^{\prime}}}\left(\aleph \right)+\left(1-\phi +\frac{{\rho }_{s}}{{\rho }_{f}}\phi \right)\left(F\left(\aleph \right){F}^{{^{\prime}}{^{\prime}}}\left(\aleph \right)-2{{F}^{{\prime}}}^{2}\left(\aleph \right)+4\lambda G\left(\aleph \right)\right)\\ &\quad-\left(1+\frac{3\left(\sigma -1\right)\phi }{\left(\sigma +2\right)-\left(\sigma -1\right)\phi }\right)H{a}^{2}{F}^{{\prime}}\left(\aleph \right)=0,\end{aligned}$$

22 $$\begin{aligned}&\left(1+7.3\phi +123{\phi }^{2}\right){G}^{{^{\prime}}{^{\prime}}}\left(\aleph \right)+\left(1-\phi +\frac{{\rho }_{s}}{{\rho }_{f}}\phi \right)\left(F\left(\aleph \right){G}^{{\prime}}\left(\aleph \right)-2{F}^{{\prime}}\left(\aleph \right)G\left(\aleph \right)-4\lambda {F}^{{\prime}}\left(\aleph \right)\right)\\ &\quad-\left(1+\frac{3\left(\sigma -1\right)\phi }{\left(\sigma +2\right)-\left(\sigma -1\right)\phi }\right)H{a}^{2}G\left(\aleph \right)=0,\end{aligned}$$

23 $$\begin{aligned}&\frac{\left(\left(1+2.72\phi +4.97{\phi }^{2}\right)+R\right)}{{\mathrm{Pr}}_{f}\left(1+3.9\phi +82.1{\phi }^{2}\right)}{\theta }^{{^{\prime}}{^{\prime}}}\left(\aleph \right)+\left(1-\phi +\frac{{\left(\rho {c}_{p}\right)}_{s}}{{\left(\rho {c}_{p}\right)}_{f}}\phi \right)F\left(\aleph \right){\theta }^{{\prime}}\left(\aleph \right)+{D}_{f}{\beta }^{{^{\prime}}{^{\prime}}}\left(\aleph \right)\\ &\quad+\left(1+7.3\phi +123{\phi }^{2}\right){Ec}_{x}\left({{F}^{{^{\prime}}{^{\prime}}}}^{2}\left(\aleph \right)+{{G}^{{\prime}}}^{2}\left(\aleph \right)\right)=0,\end{aligned}$$

24 $${\beta }^{{{\prime}}{{\prime}}}\left(\aleph \right)+ScF\left(\aleph \right){\beta }^{{\prime}}\left(\aleph \right)+SrSc{\theta }^{{^{\prime}}{^{\prime}}}\left(\aleph \right)-Sc\delta \beta \left(\aleph \right)=0.$$

Step 2. The generalized form of trial solution is:

25 $${\mathcal{ \tilde T}}\left(\aleph \right)={\mathbf{C}}_{l}^{T}\mathfrak{B}\left(\aleph \right),$$

where $\sum_{i=1}^{{2}^{k-1}}\sum_{j=0}^{M-1}{\Delta }_{ij}^{q}{\mathcal{H}}_{ij}\left(\mathrm{\aleph }\right)={\mathbf{C}}_{l}^{T}\mathfrak{B}\left(\mathrm{\aleph }\right),q=l=\mathrm{1,2},\mathrm{3,4}$ for ${\mathcal{\tilde T}}\left(\mathrm{\aleph }\right)=(\tilde{F }\left(\mathrm{\aleph }\right),\tilde{G }\left(\mathrm{\aleph }\right),{ \tilde \theta }\left(\mathrm{\aleph }\right),{\tilde \beta }\left(\mathrm{\aleph }\right))$ respectively. The column matrices ${\mathbf{C}}_{l}$ and $\mathfrak{B}\left(\mathrm{\aleph }\right)$ in Eq. (25) associated as:

$${\mathbf{C}}_{l}={\left[{\Delta }_{1,k}^{i}\right]}^{T}, k=\mathrm{0,1},2,\dots ,$$

Trial solutions Eq. (25) can be rewritten as:

$${\mathcal{\tilde T}}\left(\aleph \right)={{\varvec{\Lambda}}}_{l}^{T}{\varvec{\upchi}}\left(\aleph \right),{\varvec{\upchi}}\left(\aleph \right)={\left[1,\aleph ,{\aleph }^{2},{\aleph }^{3},\dots \right]}^{T}$$

In above the matrices ${{\varvec{\Lambda}}}_{l}$ can be observe in^34–36^. The above trial solutions following as:

26 $${\mathcal{\tilde T}}\left(\aleph \right)=\sum_{n=0}^{M}{\tilde{d }}_{n}^{l}{\aleph }^{n}.$$

Step 3. Residuals of equations (temperature, velocities and concentration) are achieved:

$$\begin{aligned}&{\mathbf{R}}_{F}=\left(1+7.3\phi +123{\phi }^{2}\right){\tilde{F }}^{{{\prime}}{{\prime}}{{\prime}}}\left(\aleph \right)+\left(1-\phi +\frac{{\rho }_{s}}{{\rho }_{f}}\phi \right)\left(\tilde{F }\left(\aleph \right){\tilde{F }}^{{{\prime}}{{\prime}}}\left(\aleph \right)-2{{\tilde{F }}^{{{\prime{2}}}}}\left(\aleph \right)+4\lambda \tilde{G }\left(\aleph \right)\right)\\ &\quad-\left(1+\frac{3\left(\sigma -1\right)\phi }{\left(\sigma +2\right)-\left(\sigma -1\right)\phi }\right)H{a}^{2}{\tilde{F }}^{{\prime}}\left(\aleph \right),\end{aligned}$$

$$\begin{aligned}{\mathbf{R}}_{G}&=\left(1+7.3\phi +123{\phi }^{2}\right){\tilde{G }}^{{{\prime}}{{\prime}}}\left(\aleph \right)+\left(1-\phi +\frac{{\rho }_{s}}{{\rho }_{f}}\phi \right)\left(\tilde{F }\left(\aleph \right){\tilde{G }}^{{\prime}}\left(\aleph \right)-2{\tilde{F }}^{{\prime}}\left(\aleph \right)\tilde{G }\left(\aleph \right)-4\lambda {\tilde{F }}^{{\prime}}\left(\aleph \right)\right)\\ &\quad-\left(1+\frac{3\left(\sigma -1\right)\phi }{\left(\sigma +2\right)-\left(\sigma -1\right)\phi }\right)H{a}^{2}\tilde{G }\left(\aleph \right),\end{aligned}$$

$${\mathbf{R}}_{\theta }=\frac{\left(\left(1+2.72\phi +4.97{\phi }^{2}\right)+R\right)}{{\mathrm{Pr}}_{f}\left(1+3.9\phi +82.1{\phi }^{2}\right)}{ \tilde \theta }^{{{\prime}}{{\prime}}}\left(\aleph \right)+\left(1-\phi +\frac{{\left(\rho {c}_{p}\right)}_{s}}{{\left(\rho {c}_{p}\right)}_{f}}\phi \right)\tilde{F }\left(\aleph \right){ \tilde \theta }^{{\prime}}\left(\aleph \right)+{D}_{f}{\tilde \beta }^{{{\prime}}{{\prime}}}\left(\aleph \right)+\left(1+7.3\phi +123{\phi }^{2}\right){Ec}_{x}\left({{\tilde{F }}^{{{{\prime}}{{\prime{2}}}}}}\left(\aleph \right)+{{\tilde{G }}^{{{\prime{2}}}}}\left(\aleph \right)\right),$$

$${\mathbf{R}}_{\beta }={\tilde \beta}^{{{\prime}}{{\prime}}}\left(\aleph \right)+Sc\tilde{F }\left(\aleph \right){\tilde \beta}^{{\prime}}\left(\aleph \right)+SrSc{\tilde \theta}^{{{\prime}}{{\prime}}}\left(\aleph \right)-Sc\delta {\tilde \beta }\left(\aleph \right).$$

Step 4. In order to explore the unknown constants ${\tilde \Delta }$’s apply the Galerkin concept. We got the following system of nonlinear equations in generalized form as:

$${\mathcal{\bf K}}_{{b}}^{n}={\int }_{0}^{{\aleph }_{\infty }}{\mathbf{R}}_{{b}}\frac{\mathrm{d}}{\mathrm{d}{\tilde \tau }_{n}^{l}}{{\tilde b}}\left(\aleph \right)\mathrm{d}\aleph , n=\mathrm{1,2},\dots ,{2}^{k-1}M-3,$$

where ${b}=\left(F,G,\theta ,\beta \right),$ ${\mathcal{\bf K}}_{{b}}^{n}=\left({\mathbf{E}}_{F}^{n},{\mathbf{E}}_{G}^{n},{\mathbf{E}}_{\theta }^{n},{\mathbf{E}}_{\beta }^{n}\right), {\mathbf{R}}_{{b}}=({\mathbf{R}}_{F},{\mathbf{R}}_{G},{\mathbf{R}}_{\theta },{\mathbf{R}}_{\beta })$ and $l=\mathrm{1,2},\mathrm{3,4}$ for $F,G,\theta ,$ and $\beta$ respectively.

**Figure 2:**
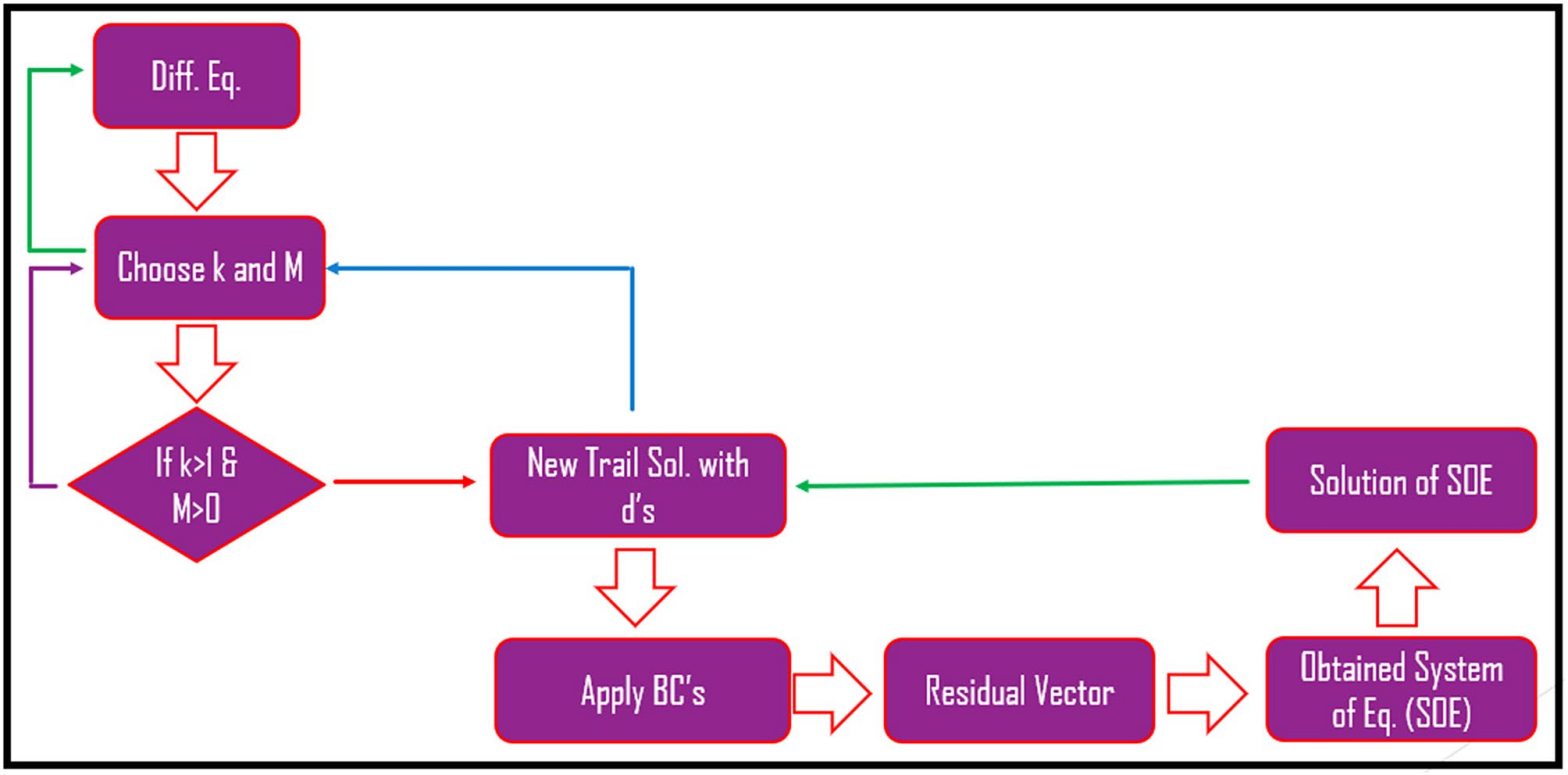
Flow chart of wavelets method.

Step 5. The values of ${\tilde \Delta }$’s (unknowns) is achieved. Consequently, the values of $\Delta$’s accomplished by inserting the unknowns ${ \tilde \Delta }$’s. There is complete flow chart of the numerical strategy is given in Fig. 2.

## Results and discussion

**Figure 3:**
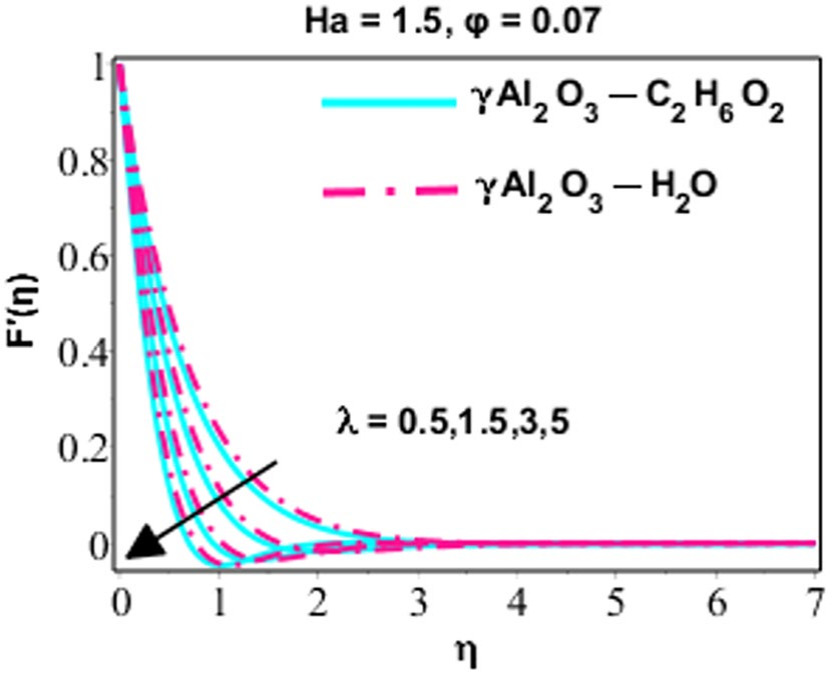
Behavior of ${F}^{{\prime}}\left(\eta \right)$ due to the variation in $\lambda$.

**Figure 4:**
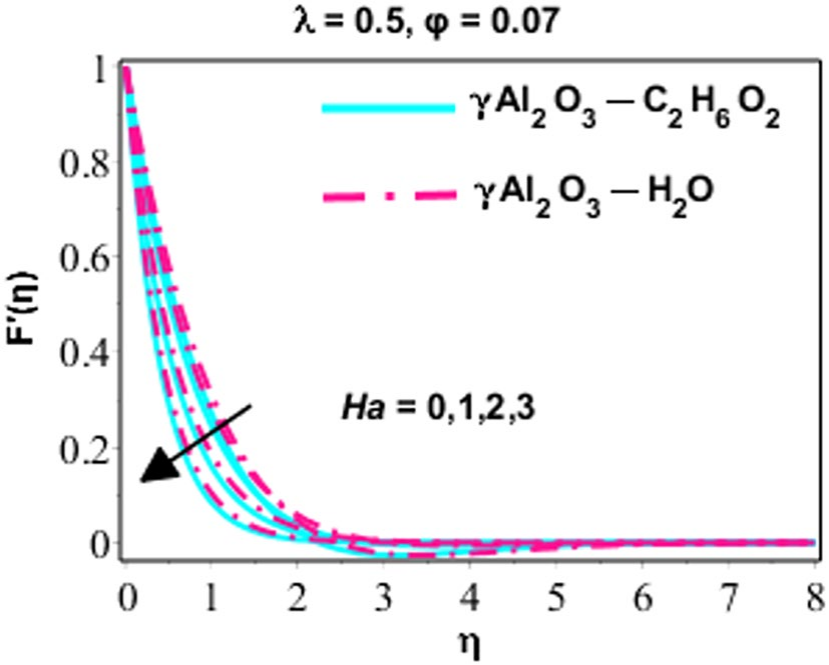
Character of ${F}^{{\prime}}\left(\eta \right)$ as varying $Ha$.

**Figure 5:**
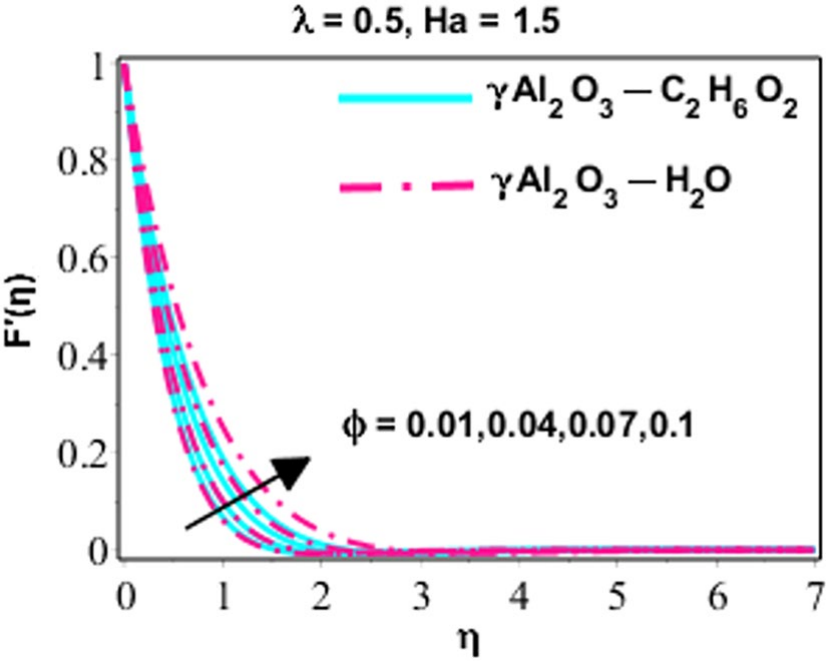
Character of ${F}^{{\prime}}\left(\eta \right)$ as varying $\phi$.

**Figure 6:**
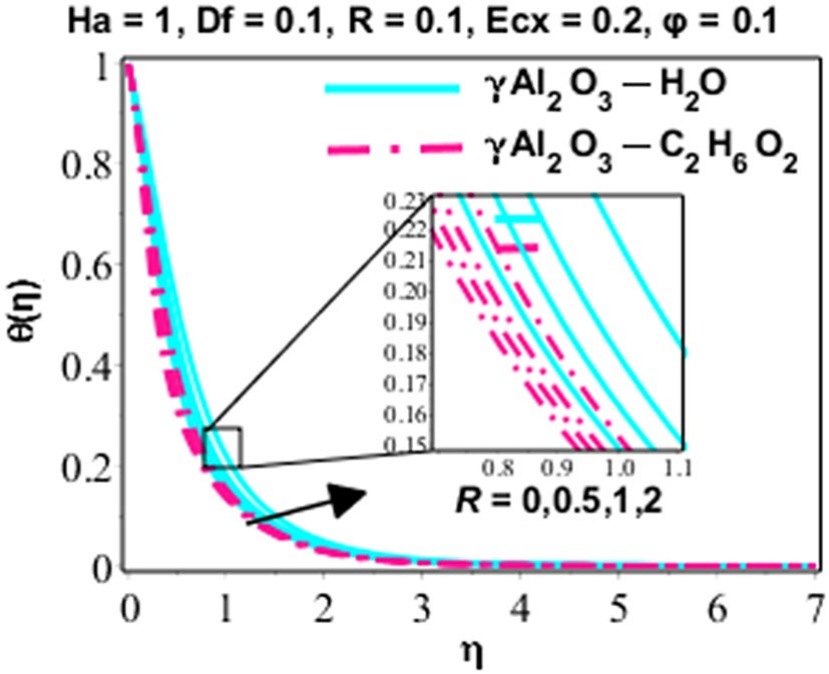
Character of $\theta \left(\eta \right)$ due to the variation in $R$.

**Figure 7:**
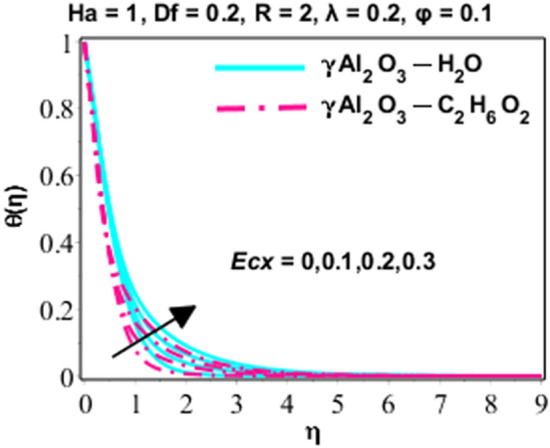
Character of $\theta (\eta )$ due to the variation in $E{c}_{x}$.

**Figure 8:**
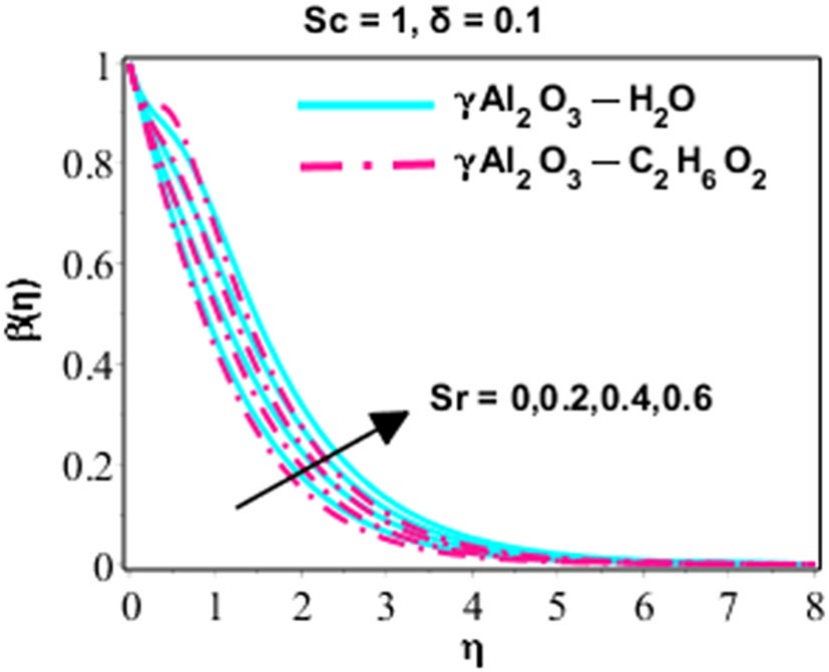
Character of $\beta \left(\eta \right)$ due to the variation in $Sr$.

**Figure 9:**
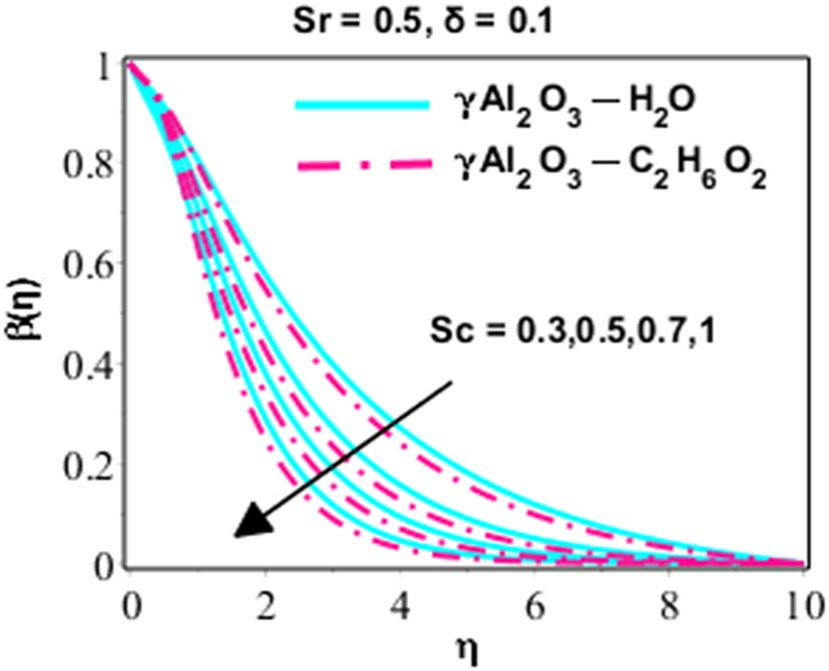
Character of $\beta \left(\eta \right)$ due to the variation in $Sc$.

Effects of Dufour and Soret on heat and mass transmission of rotating flow of $\upgamma {\mathrm{Al}}_{2}{\mathrm{O}}_{3}-{\mathrm{C}}_{2}{\mathrm{H}}_{6}{\mathrm{O}}_{2}$ and $\upgamma {\mathrm{Al}}_{2}{\mathrm{O}}_{3}$–water nanofluids with MHD and thermal radiation, and Eckert numbers. The problem is modelled in terms if partial differential equations (PDEs) with associated physical conditions which is then transformed into ODEs, in Eqs. (13)–(20) along with BCs and solved by proposed approach and parametric studies relevant to physics of problem are presented in this portion of paper. Plotted Figs. 3, 4, 5, 6, 7, 8 and 9 shows the enhancement of various parameters for flow, thermal mechanism and concentration of solute in nano-fluid. It has already been deliberated in the earlier section that two categories of nano fluid i.e. $\gamma {\mathrm{Al}}_{2}{\mathrm{O}}_{3}{-}{\mathrm{H}}_{2}\mathrm{O}$ and $\gamma {\mathrm{Al}}_{2}{\mathrm{O}}_{3}-{\mathrm{C}}_{2}{\mathrm{H}}_{6}{\mathrm{O}}_{2}$ is incorporated to study the modelled situation of the problem. In this section, we use $\aleph =\eta$ just for simplicity.

Figures 3, 4 and 5 are sketched performance of dimensionless $x$ and $y$ components of velocity for different parameters like $Ha$ (Hartmann number), $\phi$ (volume fraction) and $\lambda$ (rotation number). Attitude of $x-$ component flow in nano-fluid ($\gamma {\mathrm{Al}}_{2}{\mathrm{O}}_{3}{-}{\mathrm{H}}_{2}\mathrm{O}$ and $\gamma {\mathrm{Al}}_{2}{\mathrm{O}}_{3}-{\mathrm{C}}_{2}{\mathrm{H}}_{6}{\mathrm{O}}_{2}$) for variated values of $\lambda$ is explained in Fig. 3. The observations display that the velocity of nano-fluid is decreasing gradually between $0\ge \eta \le 3$ and $\gamma {\mathrm{Al}}_{2}{\mathrm{O}}_{3}-{\mathrm{C}}_{2}{\mathrm{H}}_{6}{\mathrm{O}}_{2}$ has higher velocity as compared $\gamma {\mathrm{Al}}_{2}{\mathrm{O}}_{3}{-}{\mathrm{H}}_{2}\mathrm{O},$ this is because of boundary layer thickness become thinner with increasing values of $\lambda$ which promote the decreasing behavior of velocity of nano-fluid. However, it just can be observed between $0\ge \eta \le 3$ and after this no variation can be perceived for growing values of $\lambda .$ On the other hand the velocity of the system is monotonically declining for lesser values of $\lambda$ and this approach become non-monotonic for $\lambda =1.5, 3, 5$ and $0\ge \eta \le 3$. Actually, owing to rotation effects oscillatory motion of the fluid close to the wall is acted and for $\lambda \ge 1.5$ the rotation properties become dominant as associated to extending effects. From this we can conclude that for minor values of $\lambda (=1.5)$ the velocity is monotonically falling and higher values of $\lambda (=1.5, 3.0, 5.0)$ area adjacent to wall show oscillatory design. In the next behavior of $x-$components flow for $\left(Ha\right)$ is illustrated in Fig. 4. Increasing behavior of $f{^{\prime}}(\eta )$ for increasing values of solid volume fraction is studied by Fig. 5. In all these figures, (Figs. 3, 4, and 5), it is very important to note that the velocity profiles for $\gamma {\mathrm{Al}}_{2}{\mathrm{O}}_{3}{-}{\mathrm{H}}_{2}\mathrm{O}$ is higher than $\gamma {\mathrm{Al}}_{2}{\mathrm{O}}_{3}-{\mathrm{C}}_{2}{\mathrm{H}}_{6}{\mathrm{O}}_{2}$, indeed it is due to the fact that $\gamma {\mathrm{Al}}_{2}{\mathrm{O}}_{3}-{\mathrm{C}}_{2}{\mathrm{H}}_{6}{\mathrm{O}}_{2}$ is more viscous than $\gamma {\mathrm{Al}}_{2}{\mathrm{O}}_{3}{-}{\mathrm{H}}_{2}\mathrm{O}$ nanofluid, and hence, due to high viscosity, the velocity profiles is lower.

Special effects of temperature in dimensionless form are represented in Figs. 6 and 7 for various parameters like radiation and Eckert number. The effects of $R$ on heat energy are explained in Fig. 6. Figure 6 is clarifying that graph of heat energy is increasing because the applied radiations provide more energy to nanoparticles which become the reason of growing temperature. The thickness of TBL of $\gamma {\mathrm{Al}}_{2}{\mathrm{O}}_{3}-{\mathrm{C}}_{2}{\mathrm{H}}_{6}{\mathrm{O}}_{2}$ is lesser than that of $\gamma {\mathrm{Al}}_{2}{\mathrm{O}}_{3}{-}{\mathrm{H}}_{2}\mathrm{O}.$ Rising manners of the thermal mechanism are perceived for altered values of Eckert number in Fig. 7. It is clear from the mathematical form of the physical model that Eckert number has direct relation versus viscous dissipation and growing values of Eckert number become the cause to boost up the viscous dissipation and finally fluid temperature is enhanced.

Graphical analysis of concentration of solute with variated values of different parameter like Schmidt and Soret number which are elaborated in the considered model of earlier section of this paper. Soret number and diffusion process have directly proportional relation between them. Due to this relation, increasing attitude can be seen in Fig. 8 for concentration of solute with growing values of Soret number. In Figs. 6, 7, 8 and 9, it is noted that $\gamma {\mathrm{Al}}_{2}{\mathrm{O}}_{3}-{\mathrm{C}}_{2}{\mathrm{H}}_{6}{\mathrm{O}}_{2}$ temperature and concentration profiles are lower than γAl2O3 − H2O. It is due to the fact that the structure of ethylene glycol (${\mathrm{C}}_{2}{\mathrm{H}}_{6}{\mathrm{O}}_{2}$) has more sites for hydrogen bonding than water. For this reason, ethylene glycol has stronger intermolecular forces of attraction and much more viscous than water.

Decaling attitude of concentration of solute for changed prices of Schmidt number is elaborated in Fig. 9. It is clear from the relation of the Schmidt number epitomized in the former segment that Schmidt number is engaged with diffusion property of solute by inversely proportional relation. So that is why, diffusion procedure is displayed opposite behavior with developing values of Schmidt number. From all these figures, it is noted that he asymptotic convergence of all boundary layer profiles is appreciated but free stream length is varying for these figures, indeed, it depends on the specific values of the selected parameters, and in particular the effect of that parameter to which the variation is given. In general, the asymptotic convergence for each figure is different.

Table 2 demonstrates thermal (properties) of ethylene, pure water and Alumina. Tables 3 and 4 are created to validate the assessment of the attained outcomes of ${F}^{{{\prime}}{{\prime}}}\left(0\right)$ and ${G}^{{\prime}}\left(0\right)$ obtain by means of the proposed method. These tables are evidenced that the proposed approach is accurate and useful to solve desired problem (Eqs. 13–20).

**Table 2:** Thermophysical properties of ethylene, water and Alumina.

	$\rho \left(\mathrm{kg }{\mathrm{m}}^{-3}\right)$	${c}_{p}$	$k\left(\frac{W}{m}\right)K$	$\beta$	$\mathrm{Pr}$	$\sigma$
${\mathrm{H}}_{2}\mathrm{O}$	998.3	4182	0.60	20.06	6.96	5.5 × 10^–6^
${\mathrm{C}}_{2}{\mathrm{H}}_{6}{\mathrm{O}}_{2}$	1116.6	2382	0.249	65	204	35 × 10^5^
${\mathrm{Al}}_{2}{\mathrm{O}}_{3}$	3970	795	40	0.85	-	1.1 × 10^–6^

**Table 3:** Assessment of the numerical values of ${-F}^{{^{\prime}}{^{\prime}}}\left(0\right)$ achieved by means of modified Chebyshev wavelets method with already existing results^21,40^ when $M=0.$

$\lambda$	Error with^41^	Error with^21^	Error with RK
0.2	${10}^{-4}$	${10}^{-6}$	${10}^{-6}$
0.5	${10}^{-5}$	${10}^{-6}$	${10}^{-6}$
2	${10}^{-4}$	${10}^{-6}$	${10}^{-6}$
5	${10}^{-4}$	${10}^{-6}$	${10}^{-6}$
10	${10}^{-4}$	${10}^{-5}$	${10}^{-5}$
50	${10}^{-3}$	${10}^{-4}$	${10}^{-6}$

**Table 4:** Validation of the numerical outcomes of $-G{^{\prime}}\left(0\right)$ shear stress achieved by means of modified Chebyshev wavelets method with already existing results^21,40^ when $M=0.$

$\lambda$	Error with^40^	Error with^21^	Error with RK
0.2	${10}^{-6}$	${10}^{-7}$	${10}^{-6}$
0.5	${10}^{-6}$	${10}^{-7}$	${10}^{-6}$
2	${10}^{-5}$	${10}^{-6}$	${10}^{-5}$
5	${10}^{-5}$	${10}^{-6}$	${10}^{-5}$
10	${10}^{-4}$	${10}^{-5}$	${10}^{-4}$
50	${10}^{-3}$	${10}^{-5}$	${10}^{-5}$

## Conclusion

The aspects of Dufour, **S**oret, viscous dissipation and thermal radiation in the flow of $\upgamma {\mathrm{Al}}_{2}{\mathrm{O}}_{3}{-}{\mathrm{H}}_{2}\mathrm{O}$ and $\upgamma {\mathrm{Al}}_{2}{\mathrm{O}}_{3}-{\mathrm{C}}_{2}{\mathrm{H}}_{6}{\mathrm{O}}_{2}$ over an exponential moveable surface are captured. The current model in view of mass and heat phenomena are simulated by new scheme called Chebyshev wavelets method. The key findings are simulated below:

- Results demonstrated that the proposed approach is excellent, accurate and useful to capture the numerical results.
- Velocity profiles in view of $\gamma {\mathrm{Al}}_{2}{\mathrm{O}}_{3}-{\mathrm{C}}_{2}{\mathrm{H}}_{6}{\mathrm{O}}_{2}$ nanofluid are lower than the $\gamma {\mathrm{Al}}_{2}{\mathrm{O}}_{3}{-}{\mathrm{H}}_{2}\mathrm{O}$ nanofluid.
- Temperature and concentration profiles are dominant when $\gamma {\mathrm{Al}}_{2}{\mathrm{O}}_{3}{-}{\mathrm{H}}_{2}\mathrm{O}$ nanofluid is considered.
- The viscosity of $\gamma {\mathrm{Al}}_{2}{\mathrm{O}}_{3}-{\mathrm{C}}_{2}{\mathrm{H}}_{6}{\mathrm{O}}_{2}$ is higher than $\gamma {\mathrm{Al}}_{2}{\mathrm{O}}_{3}{-}{\mathrm{H}}_{2}\mathrm{O}.$

## Future scope of the study

In this work, we have considered viscous fluid model of nanofluid with two types of base fluids (${\mathrm{C}}_{2}{\mathrm{H}}_{6}{\mathrm{O}}_{2} \mathrm{and} {\mathrm{H}}_{2}\mathrm{O}$). In future, this work can be extended for other non-Newtonian fluids, such as rate types fluid, differential type fluids etc. As for numerical simulations we have used an extended version of wavelets scheme-based Chebyshev polynomials, therefore, in future one may develop a stronger and faster technique for numerical simulations.
